# Delayed Gel Indurations as an Adverse Effect of Polyacrylamide Filler and Its Easy Treatment

**DOI:** 10.1155/2012/539153

**Published:** 2012-10-09

**Authors:** Hossein Kavoussi, Ali Ebrahimi

**Affiliations:** Hajdaie Dermatology Clinic, Kermanshah University of Medical Sciences (KUMS), Golestan Avenue, Kermanshah 6714653113, Iran

## Abstract

*Background*. The more increasing use of permanent soft tissue fillers such as polyacrylamide hydrogel (PAAG) for aesthetic purposes, the more adverse events resulting from them are reported. Occasionally, nonserious complications and misdiagnosis result in unnecessary surgeries and sequels. *Objective*. To introduce delayed gel indurations (DGIs) as a late onset complication of PAAG and its easy treatment. *Patient and Methods*. Twenty patients (17 females and 3 males) referred to us with subcutaneous mass at injected site of PAAG. We diagnosed DGI based on clinical and sonography findings and treatment was performed with a hole by 16-gauge needle and squeezing. *Results*. From 20 patients with 21 cases of DGI, 5 (23.8%), 5 (23.8%), and 5 (23.8%) cases in cheeks, glabella, and lips were seen, respectively. The time range between PAAG injection and presentation of patients was 10–28 months (mean = 17.5%). All of the patients responded very well to treatment without recurrence and any complications. *Conclusion*. DGI is a nonserious, late onset, and easily treated complication of PAAG that is probably induced due to water exchange between gel and surrounding tissue and modest host immune reaction to gel.

## 1. Introduction 

In recent years, injectable filler is a common and noninvasive cosmetic procedure that is used for skin defect, facial wrinkle and folds, and depressed scars. The perfect filler should be low-priced, safe, and induce long-term effects [1].

 Soft tissue augmentation can be classified according to different criteria, one of which is longevity that includes temporary, semipermanent, and permanent [2].

Biodegradable hyaluronic acid and collagen are considered as temporary fillers and last less than 9 months, and because of their short-lived side effects, they should always be taken into account as the first line of therapy [2–4].

Semipermanent fillers such as fat, Sculptra (composed of poly-L-lactic acid microspheres, sodium carboxymethylcellulose, and nonpyrogenic mannitol), and Radiesse (composed of 30% calcium hydroxylapatite microspheres suspended in an aqueous gel carrier) are partially biodegradable and last 1 to 3 years. The best cosmetic effects of these fillers are preserved with annual touch-up [2, 3].

Nonreversible and nonbiodegradable fillers with very long duration such as silicone oil, polyacrylamide hydrogel and polymethylmethacrylate microspheres suspended in noncrosslinked collagen have been developed for facial augmentation [5, 6].

Polyacrylamide hydrogel (PAAG) is permanent filler that contains 2.5% polyacrylamide and 97.5% water which has been used for facial corrective surgery and breast voluming worldwide for many years [7–9].

Although some studies have indicated PAAG as a well-tolerated product with desirable aesthetic results and a few complications [10, 11], many studies have shown that numerous adverse events occur after using this permanent filler. This complication may be, transient, nonsignificant (pain, hematoma), surgical route error (irregularity, gel accumulation, asymmetry), infectious (abscess), host tissue reaction (foreign body granuloma, edema, inflammation, redness, sensitivity), and miscellaneous such as gel migration, lumpiness, and gel indurations [9, 11–14]. 

Among present studies, based on the findings of Wolter and Pallua [11], the rate of gel indurations adverse event was 11.2% throughout the 60 months of followup.

For the detection of the presence of temporary and permanent fillers, ultrasonography is a noninvasive and popular diagnostic tool [15].

Generally, treatment of adverse events due to PAAG is difficult [9]; thus, accurate diagnosis of the complication is very important because of performing appropriate treatment and avoiding unnecessary surgical procedures.

This study attempted to analyze and easily treat delay gel indurations (DGIs) which is one of the relatively common adverse events of PAAG injection in the facial area. 

## 2. Method 

Twenty patients (17 females and 3 males) with the age range of 19–47 years (mean = 33.3) referred to our clinic from 2008–2011 with asymptomatic subcutaneous mass on the injection site of filler (Table 1). A number of patients in our clinic and the remaining of patients in other dermatologic clinics and offices were subjected to injected PAAG with different brands. 

Patients in whom sonography confirmation of semisolid nature of mass and history of augmentation with polyacrylamide filler were recruited but patients who were pregnant and had coagulation disorder and indurations containing vessel by sonography were excluded from the study.

Clinical examination revealed nontender, nonmobile, firm, palpable, occasionally visible, and without erythematous subcutaneous mass. 

**Figure 1:**
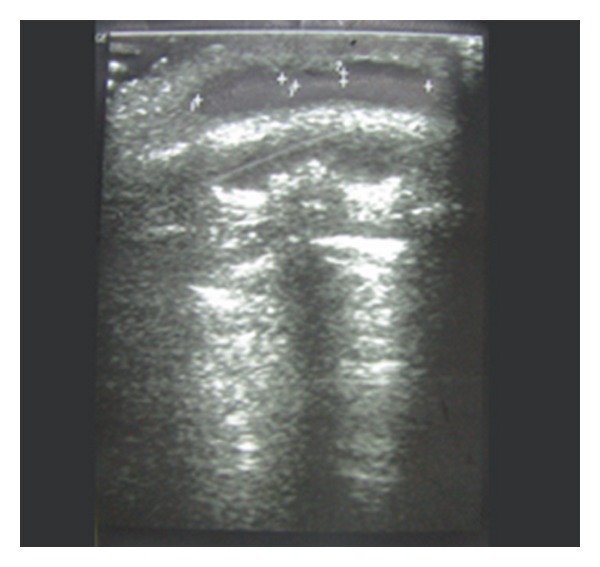
Sonogram of patient shows hypoecho subcutaneous mass.

To find the probable nature of subcutaneous mass and to rule out other pathologic mass, ultrasonography was done in all patients. Sonography findings included well-defined hypoecho subcutaneous masses, and Doppler study indicated avascularity that was compatible with semisolid nature of mass (Figure 1).

**Figure 2:**
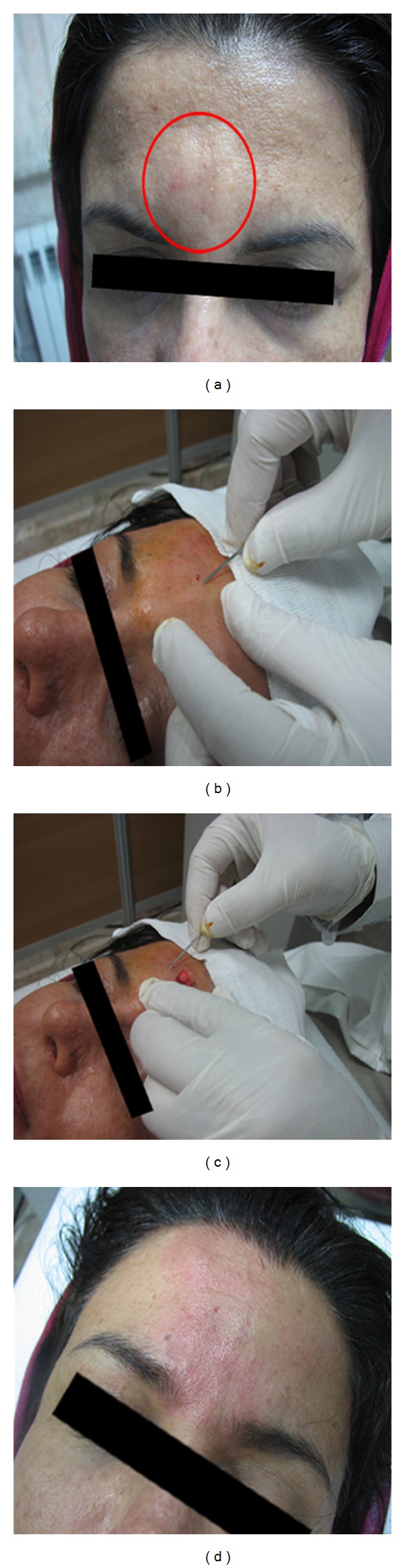
(a) Patient with delayed gel indurations in glabella area. (b) Using needle (gauge 16) induced hole and tunneling. (c) Squeezing or sliding pressure results in drainage of remaining of gel. (d) Complete exhaust gel.

Patients were employed in our study by giving information and obtaining written consent. For treatment of DGI, first subcutaneous mass was determined and marked in upright position; injection of local anesthesia (lidocaine 2% + epinephrine 1/1000) or nerve block was done. Then, using a needle (gauge 16) an opening or hole and tunneling were induced. Finally, 2-3 times sliding pressure or squeezing was done that resulted in complete drainage and disappearance of DGI (Figures 2(a)–2(d)).

 Because of the semisolid nature of drainage material, pathologic evaluation was not possible, but it was subjected to microbiology study. We recommended postoperative continuous and moderate pressure on the site of DGI for 24–48 hours.

The proposal of the study was approved by the Ethics Committee of Kermanshah University of Medical Sciences and registered in IRCT database.

## 3. Results

From 20 patients with 21 cases of DGI, 5 (23.8%), 5 (23.8%), 5 (23.8%) cases in cheeks, glabella and lips, 4 (19%) cases in nasolabial fold and 2 (9.5%) in lower lid were seen. In one patient, concurrent showed DGI in both cheeks and lower lid (Table 1). 

The time range between injection and induced DGI or presentation of patients to us was 10–28 months (mean = 17.5). Also in 7 (35%) patients, this time range was more than 20 months (Table 1).

Although 19 (90%) cases responded in one session of treatment that resulted in compete drainage of DGI but in one case, lips and cheeks involvement required second and third sessions of treatment, respectively.

In all of the cases, drainage material was semisolid, semitranslucent, and odorless; also culture of containing DGI did not show any microbial growth.

Postoperation assessment did not show any complications such as: infection, bruise, abscesses, hematoma formation, recurrence of indurations, and foreign body granuloma.

Followups of patients were associated with satisfaction of all of them and were not necessary for additional intervention. 

## 4. Discussion 

According to the widespread use of fillers for cosmetic purposes and their constantly increasing adverse events especially PAAG, notable cases of DGI as a complication of PAAG were presented to us and were easily treated without any complications and sequels.

The host response to PAAG injection can be a slight cellular reaction or occasionally vessel ingrowth that may result in fibrovascular network. Also, long-standing events consist of exchange of water between hydrogel and surrounding tissue but do not cause fibrosis [8].

Ono et al. [9] reported 10 cases of complication after PAAG filler in face area. They explained indurations in 2 cases in lower eyelid. The time intervals between injection and visit were in one case 6 and in another 0.5 months. Both cases were subjected to conservative operative method.

In a study, gel indurations/blebs were found as a complication in 9 patients (13 adverse events) that were presented as asymmetric and palpable invisible subcutaneous nodes. Almost half of them underwent gel removal by incision or needle aspiration [11].

Kalantar-Hormozi et al. [12] evaluated 542 patients receiving PAAG filler. Occurrences of lumpiness as adverse effects were seen in 5 (0.9%) cases that had been subjected to gel removal in operating room.

In a study from China, in 15 patients that experienced complication following permanent PAAG injection, 12 cases (80%) showed nodules formation [13].

Although adverse events such as lumpiness, gel-indurations, and nodule look like DGI, DGI can be different from the listed complications because of time onset, mechanism, and clinical findings. These side effects, other than DGI, are usually induced due to injection technical error, for example, amount and depth of injected filler.

We think that the mechanism of DGI may be due to water exchange between hydrogel filler and surrounding tissue, insensible host reaction to PAAG, and less likely latent infection in adjacent area such as sinusitis or dental infection. Also DGI is manifested as palpable and occasionally visible, firm, subcutaneous mass without erythema and tenderness that patients notice at least several months after injection.

In general, treatment of complication due to PAAG filler especially long-term reaction is complex or even impossible [9]. However, Pallua and Wolter [16] reported easy treatment of asymmetry of lips after augmentation of lips by PAAG injection through stab incision. Also large needle aspiration is recommended as an in-office and easy technique for permanent filler [17]. 

We diagnosed 20 patients with 21 cases of DGI based on clinical and sonography findings and this complication was treated by using a needle with gauge 16 through inducing a hole or pierce and tunneling and then squeezing that resulted in complete treatment without complication and scar formation.

In conclusion, with regard to the increasing use of fillers and their rising complications, it seems PAAG is safe filler, but it is better to be used in limited volume for skin defects and depressed scars. To reduce adverse events, we suggest this filler be used with caution in case of being used with other cosmetic procedures, higher volume and cosmetic face augmentation. Injection of PAAG with non-certified brands, presence of any infection such as dental infection and sinusitis, and inflammatory acne lesions must be avoided.

We recommend accurate diagnosis and appropriate treatment of adverse events as well as avoidance of unnecessary surgical procedures. 

## Figures and Tables

**Table 1:** Characteristics of patients.

Variables	Variables classes	Numbers	Percent
Sex			
	Female	17	85%
	Male	3	25%
Age			
	<20	1	5%
	20–29	5	25%
	30–39	10	50%
	>40	4	25%
Time onset of indurations			
	<20	13	65%
	>20	7	35%
Site involvement			
	Cheeks	5	23.8%
	Nasolabial fold	4	19%
	Glabella	5	23.7%
	Lower lid	2	9.5%
	Lips	5	23.8%
